# Ectoparasites of *Columbina passerina insularis* (Columbiformes) in the National Zoological Park, Havana, Cuba

**DOI:** 10.1590/S1984-29612021096

**Published:** 2022-01-05

**Authors:** Daniel González-Acuña, Armando Cicchino, Diana Echeverry, Karen Ardiles, Pablo Oyarzún-Ruiz, Sergey Mironov, Lucila Moreno

**Affiliations:** 1 Laboratorio de Parásitos y Enfermedades de Fauna Silvestre, Departamento de Ciencia Animal, Facultad de Ciencias Veterinarias, Universidad de Concepción, Chillán, Chile; 2 Laboratorio de Artrópodos, Departamento de Biología, Universidad Nacional de Mar del Plata, Mar del Plata, Buenos Aires, Argentina; 3 Facultad de Medicina Veterinaria, Universidad San Sebastián, Concepción, Chile; 4 Zoological Institute, Russian Academy of Sciences, Universitetskaya Quay 1, Saint Petersburg, Russia; 5 Departamento de Zoología, Facultad de Ciencias Naturales y Oceanográficas, Concepción, Chile

**Keywords:** Phthiraptera, Acari, Columbidae, ectoparasites, Phthiraptera, Acari, Columbidae, ectoparasitas

## Abstract

Ectoparasites of 18 free-living Cuban Ground Doves, *Columbina passerina insularis* (Columbiformes: Columbidae), captured in the National Zoological Park, Havana, Cuba, were identified. The collected ectoparasites included two species of lice (Phthiraptera: Ischnocera): *Columbicola passerinae* (77.1%), and *Physconelloides eurysema* (50%), as well as four species of feather mites (Astigmata: Falculiferidae): *Pterophagus lomatus* (83.3%), *Byersalges talpacoti* (50%), *Byersalges phyllophorus* (72.2%), and *Hyperaspidacarus tridentatus* (27.7%). *Pterophagus lomatus*, *B. phyllophorus*, and *H. tridentatus* represent new records for Cuba.

## Resumo

Foram identificados os ectoparasitas de 18 rolinhas-cinzentas de vida livre, *Columbina passerina insularis* (Columbiformes: Columbidae), capturadas no Parque Zoológico Nacional de Havana, Cuba. Os ectoparasitas coletados pertencem à duas espécies de piolhos (Phthiraptera: Ischnocera): *Columbicola passerinae* (77,1%) e *Physconelloides eurysema* (50%), bem como à quatro espé cies de ácaros de pena (Astigmata: Falculiferidae): *Pterophagus lomatus* (83,3%), *Byersalges talpacoti* (50%), *Byersalges phyllophorus* (72,2%) e *Hyperaspidacarus tridentatus* (27,7%). *Pterophagus lomatus*, *B. phyllophorus* e *H. tridentatus* representam novos registros para Cuba.

The Common Ground Dove, *Columbina passerina* (Linnaeus, 1758) (Columbiformes: Columbidae), has a wide distribution, spanning the southern United States (USA) to northeastern Brazil in South America. In all, 18 subspecies are recognized, with nine occurring in the Antilles (BirdLife International, 2020). Of these, the Cuban Ground Dove or “tojosa”, *Columbina passerina insularis* (Ridgway, 1888), is the most widely distributed and, in Cuba, is the most common bird of the family Columbidae (Schwartz, 1970).

Parasitological studies of *C. passerina* have previously recorded the presence of the following ectoparasites: the hippoboscid fly *Microlynchia pusilla* (Speiser, 1902) in Mexico (Tella et al., 2000); the feather mites *Pterophagus lomatus* Gaud and Barré, 1992 in Guadeloupe (Gaud & Barré, 1992a); *Byersalges phyllophorus* Gaud and Barré, 1988 in Guadeloupe, Barbados, Jamaica, Dominican Republic, and Santa Lucía (Gaud & Barré, 1988, 1992a); *Byersalges talpacoti* (Cerny, 1975) in Barbados, Guadeloupe, Jamaica, Puerto Rico, the USA, Santa Lucia, and Brazil (Atyeo & Winchell, 1984; Gaud & Barré, 1988, 1992a); and *Hyperaspidacarus tridentatus* Atyeo and Smith, 1983 in Brazil and the USA (Atyeo & Smith, 1983). The lice *Physconelloides eurysema* (Carriker, 1903) was observed in the USA, Mexico, Salvador, the Grand Cayman Islands, Colombia, Venezuela, Panama (Canal zone), and Cuba (Price et al., 1999); *Hohorstiella passerina* Hill and Tuff, 1978 in Cuba (Hill & Tuff, 1978); and *Columbicola passerinae* (Wilson, 1941) in the British West Indies, Colombia, Mexico, Venezuela, the Virgin Islands, the USA (Alabama and Texas), and Cuba (Clayton & Price, 1999).

This study provides novel information on geographical distribution of ectoparasites of the Cuban Ground Dove to further contribute to the existing knowledge about the parasites of wildlife in Cuba.

In the National Zoological Park (PZN), located west of the city of Havana (Cuba), 18 Cuban Ground Doves were captured with mist nets during September and October 2007. Ectoparasites were manually extracted and placed in 70% ethanol. Subsequently, lice were cleaned in 20% KOH and passed through ascending ethanol solutions (40%, 80%, and 100%), were rinsed for 24 h in clove oil, and finally mounted in Canada Balsam. Feather mites were rinsed in Nesbitt solution (40 g of chloral hydrate, 25 mL of distilled water, and 2.5 mL of concentrated HCl) for 72 h at room temperature and mounted on a permanent slide in Berlese’s medium (Price et al., 2003). Identification of the lice was carried out following the methods of Clayton and Price (1999) and Adams et al. (2005). The keys and species descriptions detailed by Atyeo and Smith (1983), Atyeo and Winchell (1984), Gaud and Barré (1988, 1992a, b), and Gaud and Atyeo (1996) were used for the identification of mites. For lice, the prevalence (P) was expressed as the percentage of hosts infected with 1 or more individuals of a given lice species.

In the plumage of the 18 sampled Cuban Ground Doves, 38 lice individuals (Insecta: Phthiraptera) belonging to two species, as well as feather mites representing four species of the family Falculiferidae (Acari: Astigmata), were collected.

**Figure 1:**
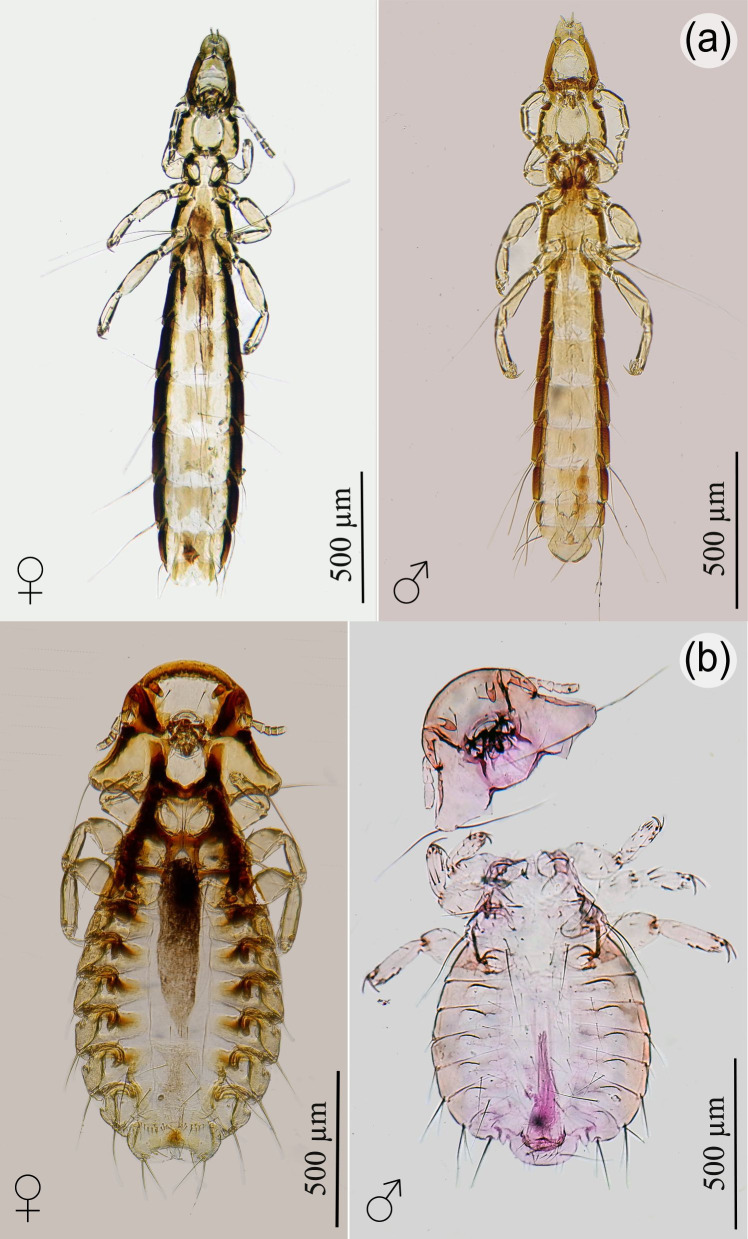
Lice collected from *Columbina passerina insularis*. (a) Dorsal view of *Columbicola passerinae* (wing lice), female and male, and (b) dorsal view of *Physconelloides eurysema* (body lice), female and male, collected from the primary and secondary wing feathers and body feathers, respectively.

Regarding the order Phthiraptera, both a wing louse, *Co. passerinae* (n=23; P=77%), and a body louse, *Ph. eurysema* (n=15; P=50%)*,* were collected from the primary and secondary wing feathers, and body feathers, respectively (Figure 1). The prevalence of lice was similar to that reported by Valim et al. (2004) for *Columbina talpacoti* (Temminck, 1810) in Brazil. Lice are permanent parasites occurring on birds with approximately 5,000 described species, of which about 3,000 are known from birds, and many more remain undescribed, particularly in the Neotropics (Price et al., 2003). The genus *Columbicola* Ewing 1929 is one of the genera with higher number of species and has approximately 88 valid species parasitizing doves and turtledoves (Price et al., 2003; Adams et al., 2005; Bush et al., 2009). Most of these species are described from these hosts from the Old World (Clayton & Price, 1999). The species *Co*. *passerinae* was described by Wilson (1941) from *C. passerina* in Auburn and Slocum (USA: Alabama), and further reported from this host by Clayton and Price (1999) in the British West Indies, Colombia, Mexico, Venezuela, the Virgin Islands, the USA (Alabama and Texas), and Cuba. This louse species was also collected from other columbids: *C. talpacoti*, *C. inca* (Lesson, 1847) (= *Scardafella inca*), *C. minuta* (Linnaeus, 1766), *C. picui* (Temminck, 1813), *Claravis mondetoura* (Bonaparte, 1856), and *Cl. pretiosa* (Ferrari-Pérez, 1886) (Tendeiro, 1965; Palma, 1973; Clayton & Price, 1999; Valim et al., 2004; Coimbra et al., 2012).

The genus *Physconelloides* Ewing, 1927 has been described as parasitizing New World columbiforms (Tendeiro, 1987) almost exclusively. Of the 30 species and subspecies described in the genus, Price et al. (1999) retained only 13 species and described three new species. *Physconelloides eurysema* was reported from six avian species of the genus *Columbina*, including *C. passerina* from Cuba (Price et al., 1999; Valim et al., 2004).

**Figure 2:**
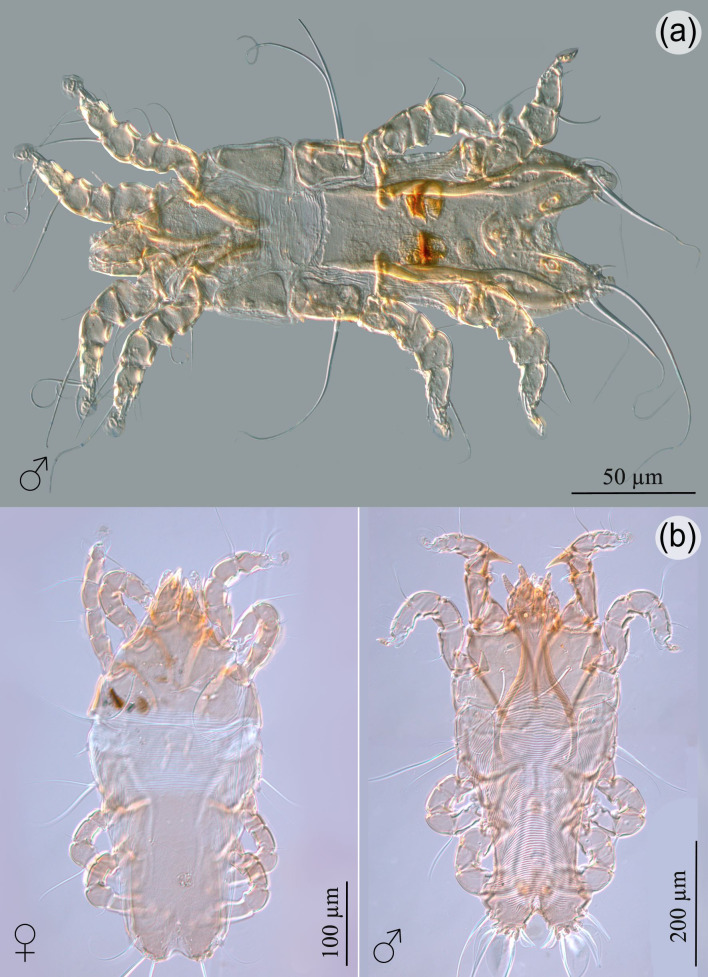
Mites collected from *Columbina passerina insularis*. (a) Dorsal view of male *Pterophagus lomatus*; (b) dorsal view of *Hyperaspidacarus tridentatus*, female and male.

**Figure 3:**
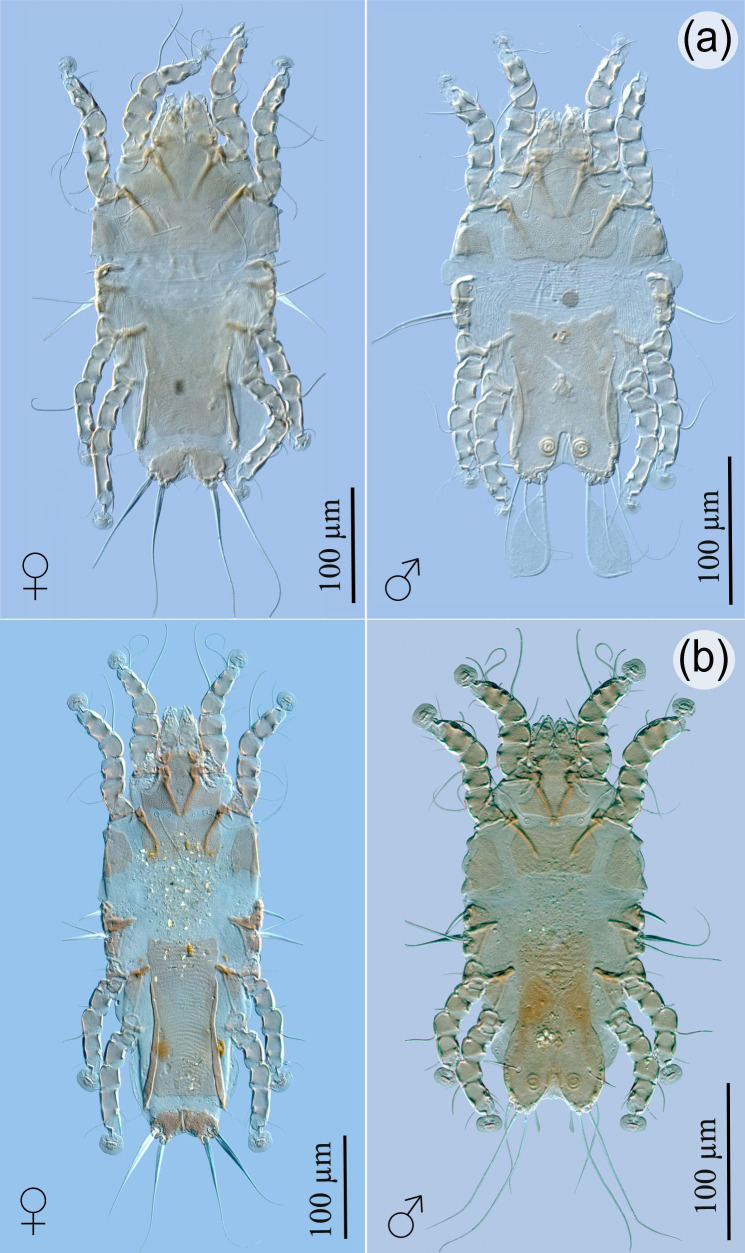
Mites collected from *Columbina passerina insularis*. (a) Dorsal view of *Byersalges phyllophorus*, female and male; (b) dorsal view of *Byersalges talpacoti*, female and male.

Feather mites were not quantified due to their high number and the fact that they could not be fully extracted. Four species belonging to the family Falculiferidae (Astigmata: Pterolichoidea) were collected: *P. lomatus* (83.3%; Figure 2a), *H. tridentatus* (27.7%; Figure 2b), *B. phyllophorus* (72.2%; Figure 3a), and *B. talpacoti* (50%; Figure 3b). *Pterophagus lomatus* was described by Gaud and Barré (1992a) from *C. passerina* in Guadeloupe, and from *C. talpacoti* in Guyana. Further, it was reported from the latter host in Brazil (Valim et al., 2004). *Hyperaspidacarus tridentatus* was formerly reported from *C. passerina* in Guadeloupe and Jamaica, and from *C. talpacoti* in Honduras, Guyana, Mexico, USA, and Brazil (Atyeo & Smith, 1983; Pedroso & Hernandes, 2016). *Byersalges phyllophorus* was described from *C. passerina* (Gaud & Barré, 1988) in Guadeloupe, and from *C. talpacoti* in Brazil (Moraes et al., 2011; Pedroso & Hernandes, 2016). *Byersalges talpacoti* was reported from four species of columbiforms in the New World: *C. passerina* in Barbados, Guadeloupe, Jamaica, Puerto Rico, Santa Lucia, and Brazil; from *C. talpacoti* in Colombia, Guyana, Suriname, and Brazil; from *C. squammata* (Lesson, 1831), *Uropelia campestris* (von Spix, 1825) and *Zenaida auriculata* (Des Murs, 1847) in Brazil; and *Z. aurita* (Temminck, 1810) and *Z. asiatica* (Linnaeus, 1758) in Cuba (Atyeo & Winchell, 1984; Gaud & Barré, 1992b; Valim et al., 2004; Goulart et al., 2011; Moraes et al., 2011; Pedroso & Hernandes, 2016).

The mites *P. lomatus*, *B. phyllophorus*, and *H. tridentatus* collected in this study are reported for the first time in Cuba. While the lice species reported in this study, although not new reports for Cuba, reaffirm the wide distribution and close of the genera *Columbicola* and *Physconelloides* with the Columbiformes.
